# Silicon-Enhanced PVA Hydrogels in Flexible Sensors: Mechanism, Applications, and Recycling

**DOI:** 10.3390/gels10120788

**Published:** 2024-12-02

**Authors:** Xiaolei Guo, Hao Zhang, Manman Wu, Zhan Tian, Yanru Chen, Rui Bao, Jinghao Hao, Xiao Cheng, Chuanjian Zhou

**Affiliations:** 1 Research Institute of Polymer Materials, School of Materials Science and Engineering, Shandong University, Jinan 250061, China; 202314126@mail.sdu.edu.cn (X.G.);; 2 School of Materials Science and Engineering, University of Jinan, Jinan 250022, China; mse_zhangh@ujn.edu.cn; 3 Weihai Research Institute of Industrial Technology, Shandong University, Weihai 264209, China

**Keywords:** dual-network, toughness, sensitive, sensors, recycle

## Abstract

Hydrogels, known for their outstanding water absorption, flexibility, and biocompatibility, have been widely utilized in various fields. Nevertheless, their application is still limited by their relatively low mechanical performance. This study has successfully developed a dual-network hydrogel with exceptional mechanical properties by embedding amino-functionalized polysiloxane (APSi) networks into a polyvinyl alcohol (PVA) matrix. This hydrogel effectively dissipates energy through dense sacrificial bonds between the networks, allowing for precise control over its tensile strength (ranging from 0.07 to 1.46 MPa) and toughness (from 0.06 to 2.17 MJ/m^3^) by adjusting the degree of crosslinking in the polysiloxane network. Additionally, the hydrogel exhibits excellent conductivity (10.97 S/cm) and strain sensitivity (GF = 1.43), indicating its potential for use in wearable strain sensors. Moreover, at the end of its life (EOL), the sensor waste can be repurposed as an adsorbent material for metal ions in water treatment, achieving the recycling of hydrogel materials and maximizing resource utilization.

## 1. Introduction

Hydrogels are three-dimensional networked crosslinked polymeric materials [1], extensively employed in fields such as drug delivery [2], medical diagnostics [3,4], strain sensors [5,6,7], and wastewater treatment [8,9,10] due to their high water absorption, excellent biocompatibility, and easily modifiable physical and chemical properties. However, traditional hydrogels often face application limitations due to their low mechanical strength and limited toughness. To overcome these limitations, various methods have been developed to enhance and toughen hydrogels, such as incorporating reinforcing materials, adjusting crosslinker types and amounts to control crosslink density, and introducing a second network to create dual-network hydrogels. Dual-network hydrogels, composed of two incompatible polymeric networks, offer a unique solution. The first network is highly crosslinked, providing mechanical strength, while the second, looser network primarily absorbs energy and enhances toughness. This structural design allows the first network to effectively disperse and transfer stress, while the second network can fracture locally, preventing total structural failure and significantly enhancing overall strength, toughness, and durability. For instance, Zhao et al. [11] developed a PAA-Al/OSA-AG dual-network hydrogel, where the first network is formed through metal coordination between PAA and Al, and the second through Schiff base crosslinking between OSA and AG, increasing the tensile strength from 34.6 kPa to 129.2 kPa. Zhang [12] and colleagues synthesized a PVA/PDMAEMA-PAAc dual-network hydrogel through a two-step process, with the first network formed by chemical crosslinking of PDMAEMA and the second by electrostatic interactions between PDMAEMA and PAA chains, exhibiting a 480% increase in compressive strength over single-network hydrogels. These studies demonstrate that dual-network hydrogels are an extremely effective strategy for enhancing the mechanical properties of hydrogels.

Wearable flexible sensors have shown immense potential in biotechnology, artificial intelligence, soft robotics, and human–machine interfaces [13,14,15,16,17,18]. Studies have shown that hydrogel-based strain sensors offer significant advantages over traditional sensors in flexibility, sensitivity, response speed, and biocompatibility, making them ideal candidates for flexible sensor fabrication [19,20,21] However, the fabrication processes for most hydrogel strain sensors are cumbersome, and their poor mechanical performance limits their application scope. Therefore, developing a simple fabrication method for hydrogel strain sensors with excellent mechanical properties is crucial for advancing technology in this field.

PVA hydrogels, known for their excellent biocompatibility, flexibility, anti-swelling properties, high water retention, and simple fabrication methods, are widely used in flexible sensors [22], biomedical applications [23], drug delivery [24], and wound dressings [25]. Although PVA hydrogels have good anti-swelling capabilities, their mechanical performance, conductivity, and adsorption capacities are relatively weak. To overcome these limitations, this study successfully developed a dual-network hydrogel with excellent overall performance by introducing amino-functionalized polysiloxane (APSi) networks into a PVA matrix. The PVA network was induced by freeze–thaw cycles, which caused partial crystallization and crosslinking of some polymer chains, forming the first network with anti-swelling properties. APSi was crosslinked with the polyacid EDTA through hydrogen bonds and ionic interactions to create the second network. The formation of the second network not only significantly enhanced the mechanical properties of the hydrogel but also endowed it with excellent heavy metal ion adsorption capability and electrical conductivity. APSi’s abundant amino and carboxyl groups strengthened the hydrogel’s ability to adsorb heavy metal ions and improved its electrical conductivity, making the hydrogel particularly advantageous for applications such as wastewater treatment and wearable strain sensors. Furthermore, the dense crosslinked structure formed by hydrogen bonds and ionic interactions between the two networks acted as sacrificial bonds to effectively absorb and dissipate external stress, thereby significantly improving the hydrogel’s tensile strength and toughness. APSi also exhibits excellent biocompatibility and chemical stability, ensuring its stability during long-term use. Therefore, the dual-network hydrogel prepared in this study shows significant improvements in both physical and chemical properties, making it an ideal material for applications in wastewater treatment and wearable sensors, with considerable potential for practical applications.

## 2. Results and Discussion

### 2.1. Characterization of APSi

The detailed synthesis procedure for APSi has been presented in the materials and methods (Figure S1). Through ^1^H NMR and FT-IR analyses, we confirmed the successful synthesis of APSi. In the ^1^H NMR spectrum, the chemical shifts and integral areas of the hydrogen signals are consistent with the structural features of the APSi side groups, confirming their presence. Additionally, in the FT-IR spectrum, the characteristic absorption peaks corresponding to NH_2_, Si–O, Si–C, and other functional groups align with the theoretical peak positions, further supporting the Si–O–Si backbone structure of APSi. The distribution of hydrogen signals in the ^1^H NMR spectrum corresponds precisely to the absorption peaks of the –OH and –NH_2_ functional groups in the FT-IR spectrum, indicating that no impurities were introduced during the synthesis of APSi and that the functional groups retained their expected structural characteristics. Overall, the characteristic peaks observed in both the ^1^H NMR and FT-IR spectra are in excellent agreement with the molecular structure of APSi, with no additional signals detected, further confirming the successful synthesis of the target product, APSi (Figure S2). Additionally, the molecular weight was verified by Gel Permeation Chromatography (GPC) tests. These results collectively demonstrate that APSi has been synthesized with the anticipated chemical structure.

### 2.2. Mechanism of Formation of PVA-APSi-EDTA Hydrogels

**Figure 1:**
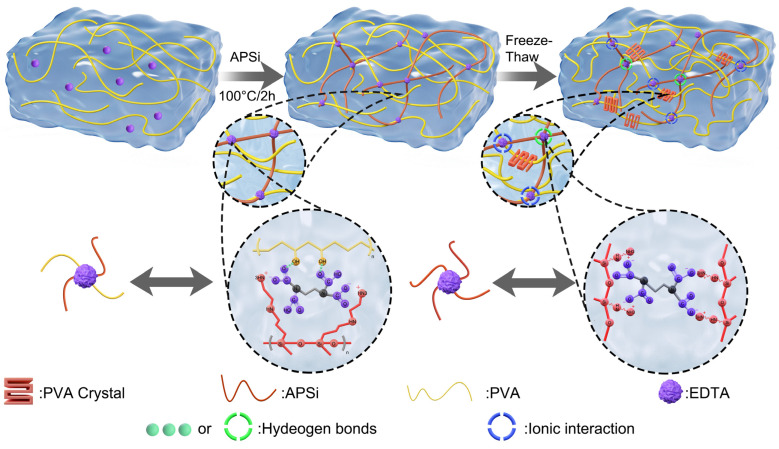
Schematic diagram of the dual-network physically crosslinked hydrogel formation.

By varying the contents of APSi and EDTA, a series of hydrogels with different network structures were prepared, the details of which are outlined in the materials and methods, and the specific compositions are listed in Table S1. Figure 1 depicts a schematic diagram of the formation of the dual-network physically crosslinked hydrogels. On one hand, PVA undergoes freeze–thaw cycles to generate crystalline domains, which act as physical crosslinking points to form a physically crosslinked PVA network [26]. On the other hand, APSi, containing –NH– and –NH_2_ groups, can crosslink with the carboxylic groups (–COOH) of EDTA through ionic interactions to form a second physical crosslinking network. Additionally, dense hydrogen bonding between the carboxylic groups of EDTA and the amino groups on APSi, as well as the hydroxyl groups on PVA, facilitates extensive interlocking between the two networks, thus forming a three-dimensional, highly crosslinked hydrogel structure [27]. In the ^1^H NMR spectrum of the PVA-APSi-EDTA_1_ swelling solution, no characteristic peaks of APSi were observed, and the FT-IR peak positions overlapped with those of distilled water (Figure S3). This indicates that APSi has been fully incorporated into the crosslinking network, and the hydrogel possesses a stable network structure.

**Figure 2:**
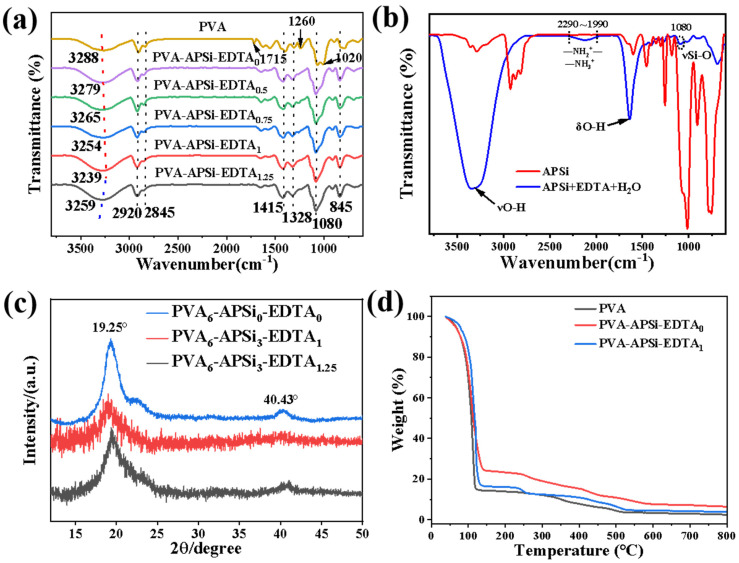
(**a**) FT-IR of PVA-APSi-EDTA hydrogels. (**b**) FT-IR of APSi and APSi/EDTA solutions. (**c**) XRD spectra of PVA, PVA-APSi-EDTA_1_, and PVA-APSi-EDTA_1.25_. (**d**) TGA of PVA, PVA-APSi-EDTA_0_ and PVA-APSi-EDTA_1_.

Based on the analysis results from XRD and FT-IR (Figure 2), the synergistic effects between PVA, APSi, and EDTA on the network structure can be further explored. The XRD pattern shows that with the addition of EDTA, the intensities of the PVA crystallization peaks gradually decrease, indicating that excessive hydrogen bonding interactions suppress the crystallization process of PVA. In the FT-IR spectrum, the shift in the OH stretching peak and the characteristic signals of the –NH_2_^+^ and –NH_3_^+^ functional groups suggest that the crosslinked network is not only formed by the physical crosslinking of PVA but also regulated by strong hydrogen bonding and ionic crosslinking between APSi and EDTA. Therefore, the interactions between PVA, APSi, and EDTA play a key role in modulating the crystallinity and network structure of the hydrogel.

### 2.3. Structure and Morphology of the Hydrogels

Figure 2a shows that with the increase in EDTA content, the –OH vibration peak at 3280 cm^−1^ shifts to a lower frequency (redshift). This shift is attributed to the formation of intra- and intermolecular hydrogen bonds among PVA chains, APSi, and EDTA, where strong hydrogen bonds reduce the force constant of the functional groups, leading to the observed redshift. When EDTA is in excess, the system displays a reduced number of hydrogen bonds, resulting in a shift to higher frequencies (blueshift) [28,29]. Peaks at 1715 cm^−1^, 1260 cm^−1^, and 1020 cm^−1^ correspond to the vibrations of ester groups C=O, and C–O antisymmetric and symmetric stretching vibrations, respectively; the introduction of APSi and EDTA along with high-temperature processes promotes the hydrolysis of residual ester groups in PVA, thereby significantly weakening the intensity of the C=O vibration peaks [30]. XRD patterns show characteristic peaks at 19.25° and 40.43° for all hydrogels, which correspond to the (100) and (110) crystal planes of PVA [31] (Figure 2c). Additionally, the peak intensity first increases and then decreases, which indicates that the introduction of APSi and EDTA increases the number of hydrogen bonds, reducing PVA’s crystalline capability and consequently weakening the intensity of the two characteristic peaks. In the PVA-APSi-EDTA_1_ sample, the influence of hydrogen bonding is most significant, causing the characteristic peak of PVA at 40.43° to nearly disappear. When EDTA is in excess, the rapid crosslinking of excess EDTA with APSi forms an uneven gel network, reducing the number of hydrogen bonds between networks, thus enhancing the crystallinity of PVA. The XRD results are consistent with the infrared findings. The FT-IR results shown in Figure 2b indicate a new peak between 1990 and 2290 cm^−1^ after APSi interacts with EDTA, corresponding to the protonation of APSi’s –NH– and –NH_2_ groups to –NH_2_^+^ and –NH_3_^+^, confirming strong ionic interactions between APSi and EDTA. In other words, the amino-functionalized polysiloxane forms a second network through ionic interactions with the polyacid EDTA. In addition, the wavenumber of the Si–OH peak is the same as the H–OH peak, and thus the signal of Si–OH is overlapped by that of H_2_O. The Si–O peak is relatively weak and locates around 1080 cm^−1^, which results from the very low content of APSi in the APSi-EDTA+H_2_O system (5 wt.%).

Figure 2d shows that the addition of APSi significantly enhanced the thermal properties of the hydrogel, as evidenced by the increased residue mass of the hydrogel. This phenomenon can be attributed to the reaction between the silicon atoms in the APSi molecules and oxygen at high temperatures, leading to the formation of a thermally stable SiO_2_ phase. In contrast, after the introduction of EDTA, the thermal performance and residue mass of the hydrogel decreased. This is because EDTA, as a polyacid, promotes the decomposition of APSi under high-temperature conditions, thereby inhibiting the formation of SiO_2_ and resulting in a decrease in thermal stability.

**Figure 3:**
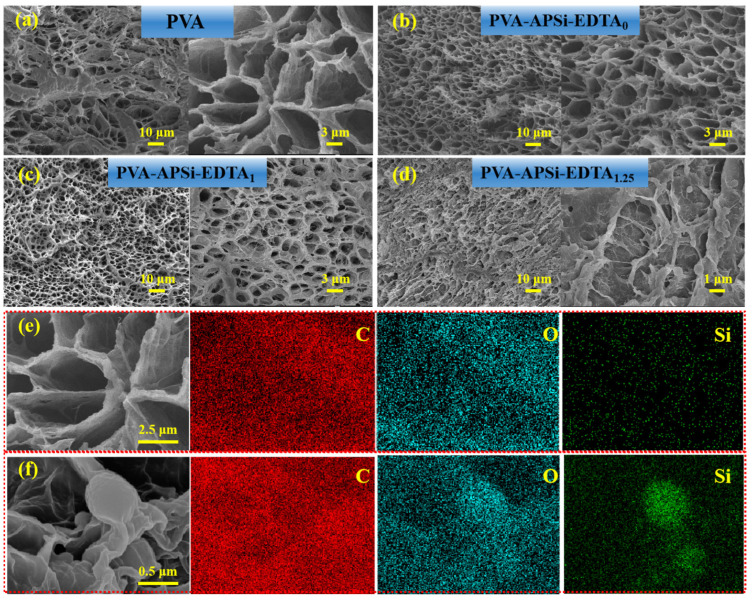
SEM images of (**a**) PVA, (**b**) PVA-APSi-EDTA_0_, (**c**) PVA-APSi-EDTA_1_, (**d**) PVA-APSi-EDTA_1.25_. (**e**) EDS image of PVA-APSi-EDTA_1_. (**f**) EDS image of PVA-APSi-EDTA_1.25_.

Observing the cross-sectional morphology of the PVA-APSi-EDTA hydrogels through SEM (Figure 3), compared to pure PVA hydrogels, the pore size is significantly reduced, and density is increased. These changes originate from the formation of the second crosslinked network made of APSi, which tightly intertwines and interlocks with PVA’s main network, enhancing the overall crosslink density [32,33]. However, when the EDTA content is too high, defects appear in the hydrogel’s microstructure: pore sizes increase and become irregular (Figure 3d). This is due to the strong ionic interactions between EDTA and APSi, causing rapid and uneven network formation when excess EDTA is present [34]. Based on the FT-IR analysis, changes in the characteristic peaks associated with EDTA can be observed, indicating that EDTA may accelerate the crosslinking reaction through strong ionic interactions with PVA and APSi. Excessive EDTA could lead to an increased crosslinking rate with APSi, resulting in the formation of an uneven network structure. This is consistent with the observed increase in pore size and irregular morphology in the SEM images. Notably, in the PVA-APSi-EDTA_1.25_ hydrogel, we observed the formation of clustered spherical structures due to the rapid crosslinking action concentrated between EDTA and APSi.

The elemental composition of PVA-APSi-EDTA_1_ and PVA-APSi-EDTA_1.25_ hydrogels is essentially consistent (Figure S4), but the elemental distribution in the PVA-APSi-EDTA_1.25_ hydrogel is uneven. In the PVA-APSi-EDTA_1.25_ hydrogel, the Si and O content is significantly elevated at the sites of the aggregated spheres, indicating the presence of localized aggregation (Figure 3f). This observation is in agreement with the SEM results, further confirming that the aggregated spheres are formed due to the rapid crosslinking reaction between excess EDTA and APSi.

By combining various characterization techniques such as ^1^H NMR, FT-IR, GPC, XRD, SEM, and EDS, this study successfully demonstrates the synthesis of PVA-APSi-EDTA hydrogel and reveals the formation process of its physical and chemical crosslinked networks. First, APSi was successfully synthesized through hydrolysis and condensation reactions, and ionically crosslinked with the amino groups of APSi and the carboxyl groups of EDTA, forming a secondary crosslinked network. Secondly, PVA formed a physical crosslinked network through freeze–thaw cycles, which tightly intertwined with the APSi-EDTA crosslinked network, thereby enhancing the structural stability of the hydrogel. Finally, the amount of EDTA significantly affects the hydrogel’s microstructure and properties. An appropriate amount of EDTA promotes the formation of the crosslinked network, while excessive EDTA may lead to uneven crosslinking, thereby influencing the hydrogel’s pore structure and mechanical properties.

### 2.4. Mechanical Properties of Hydrogels

**Figure 4:**
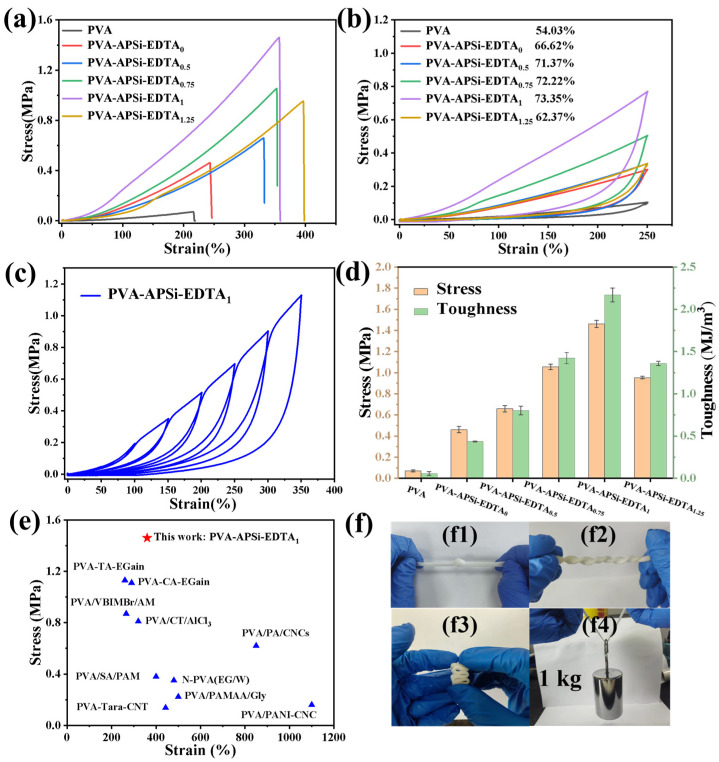
Mechanical properties of hydrogels. (**a**) Stress-strain curves during the stretching of hydrogels. (**b**) Load–unload stress–strain curves at 250% strain for hydrogels. (**c**) Cyclic load–unload stress–strain curves for PVA-APSi-EDTA_1_ hydrogel across seven strain increments. (**d**) Dual *Y*-axis graph showing the tensile strength and toughness of the hydrogels. (**e**) Comparison of the mechanical properties of PVA-APSi-EDTA_1_ hydrogel with other PVA-based hydrogels. (**f**) Knotting, twisting, stacked compression, and loading with a 1 kg weight for PVA-APSi-EDTA_1_ hydrogel (f1–f4).

The mechanical properties are crucial for assessing the quality of hydrogels. In this study, the mechanical performance of the hydrogels was evaluated through tensile testing. As illustrated in Figure 4 and Table S2, the introduction of APSi and the increase in EDTA content significantly enhanced the mechanical properties of the hydrogels. Specifically, the tensile strength increased from 0.07 MPa to 1.46 MPa, the elongation at break improved from 216% to 357%, and the toughness rose from 0.06 MJ/m^3^ to 2.17 MJ/m^3^. This exceptional mechanical performance is primarily attributed to the dual-network structure and the interactions of physical crosslinks. The second crosslinking network formed by APSi binds extensively through hydrogen bonds with the polyvinyl alcohol (PVA) network, enabling both networks to intertwine and lock together, collectively bearing external loads. Moreover, the dense hydrogen bonding and ionic interactions between networks help effectively disperse stress, reducing stress concentrations, thereby enhancing the stability and toughness of the hydrogels [35,36]. Cyclic tensile tests show that the energy dissipation efficiency initially increases and then decreases (Figure 4b), consistent with the mechanical performance of the hydrogels. Energy dissipation primarily stems from the large number of dynamic ionic interactions and hydrogen bonds acting as sacrificial bonds in the crosslinking network, allowing for a quantitative assessment of the efficacy of these sacrificial bonds. However, when the EDTA content is too high, it may lead to excessive crosslinking with APSi, resulting in an uneven network and thus a decrease in the tensile strength and toughness of the hydrogels. Overall, the PVA-APSi-EDTA_1_ hydrogel exhibits the best mechanical performance. In subsequent studies, the PVA-APSi-EDTA_1_ hydrogel will be the primary subject of research.

In the cyclic loading–unloading tests with strain increments (50–350%), the hysteresis loop of the PVA-APSi-EDTA_1_ hydrogel gradually enlarges (Figure 4c), demonstrating that as the strain increases, more dynamic physical crosslink points act as sacrificial bonds to dissipate energy during deformation, effectively preventing failure during stretching [37]. Additionally, at low strains, the hysteresis curve is almost invisible, indicating that the hydrogel maintains excellent rubber-like elastic properties under small strains. Compared to other PVA-based hydrogels, the PVA-APSi-EDTA_1_ hydrogel exhibits higher tensile strength and a significant elongation at break, reflecting its superior mechanical performance. The PVA-APSi-EDTA_1_ hydrogel also shows good flexibility, easily undergoing knotting, twisting, and stacked compression, even supporting a weight of 1 kg. These characteristics provide a solid foundation for the application of hydrogels in the field of strain sensing.

### 2.5. Electrical Properties of Hydrogels

**Figure 5:**
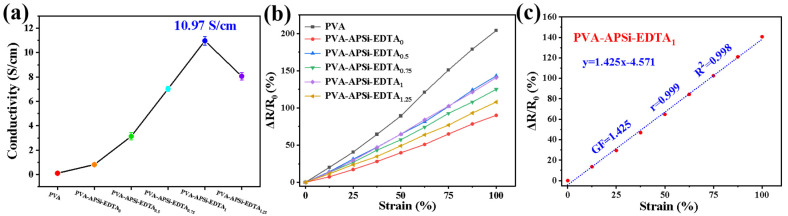
Electrical properties of hydrogels. (**a**) Conductivity of hydrogels. (**b**) Relationship between strain and relative resistance in hydrogels. (**c**) Linear relationship between strain and relative resistance in PVA-APSi-EDTA_1_ hydrogel.

Good electrical conductivity and strain sensitivity are key metrics for evaluating strain sensors. The addition of EDTA endows the hydrogel system with numerous mobile free H^+^ ions, while the porous structure of the hydrogel provides convenient pathways for the free movement of H^+^, significantly enhancing the electrical properties of the hydrogel. During the stretching of the hydrogel, the pathways for H^+^ movement are deformed or narrowed, reducing the mobility efficiency of H^+^ and consequently increasing the resistance and lowering the conductivity; when the strain is released, the pathways and routes for H^+^ gradually return to their original state, and the conductivity also recovers [38]. As shown in Figure 5a, with the incorporation of APSi and increasing EDTA content, the conductivity of the hydrogel shows an upward trend. Notably, the conductivity of the PVA-APSi-EDTA_1_ hydrogel reaches a peak of 10.97 S/cm. The conductivity of PVA-APSi-EDTA_1.25_ hydrogel is lower than that of PVA-APSi-EDTA_1_, due to the excessive EDTA disrupting the uniform gel network, which obstructs the movement of H^+^ and leads to a decrease in conductivity. Furthermore, the PVA-APSi-EDTA_1_ hydrogel not only exhibits higher conductivity but also demonstrates higher sensitivity (GF = 1.43) and a stable linear relationship between strain and relative resistance (Figure 5c, with detailed data available in Table S3). Additionally, the PVA-APSi-EDTA_1_ hydrogel’s superior mechanical properties make it an ideal candidate for further studies as a material for strain sensors.

**Figure 6:**
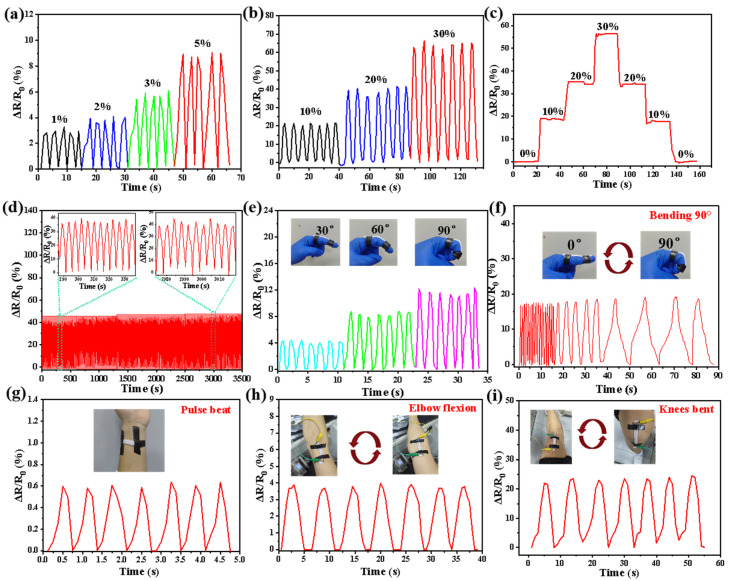
Characterization of PVA-APSi-EDTA_1_ hydrogel as a strain sensor under various dynamic loads. (**a**,**b**) Changes in relative resistance of the hydrogel under different cyclic stretching strains (1–30%). (**c**) Changes in the relative resistance of the hydrogel over time under various strains. (**d**) Relative changes in sensor resistance after soaking in water for seven days, followed by 1000 cycles of 20% strain load–unload testing; the inset shows a magnified view of the selected area. (**e**–**i**) Dynamic responses of the hydrogel strain sensor during monitoring of various human activities: (**e**) bending of fingers to varying degrees, (**f**) bending of fingers at different speeds, (**g**) pulse beating, (**h**) flexion of the elbow joint, (**i**) flexion of the knee joint.

To further explore the potential applications of PVA-APSi-EDTA_1_ hydrogel in the field of strain sensing, this study comprehensively evaluated its electrical sensitivity and stability under various cyclic stretching conditions. As depicted in Figure 6a, the PVA-APSi-EDTA_1_ hydrogel sensitively responds to minor strain signals, displaying high sensitivity to slight movements. Figure 6a–c further reveal that regardless of the magnitude of strain, the hydrogel exhibits good cyclic stability and temporal stability. Even after soaking in water for seven days and undergoing 1000 cycles of 20% strain loading and unloading, the PVA-APSi-EDTA_1_ hydrogel still shows an excellent repeatable stable response (Figure 6d), indicating that strain sensors based on this material not only have high sensitivity but also potential for long-term use [39,40]. The stability and sensitivity of this hydrogel also make it suitable for underwater sensor applications.

Next, this study will attach the PVA-APSi-EDTA_1_ hydrogel to the human body, monitoring changes in relative resistance to detect human movement. Figure 6e,f show the changes in resistance signals during the bending of a finger; as the bending angle increases, the resistance value gradually rises, consistent with the increase in elongation of the hydrogel during bending. The speed of finger bending aligns with the timing of resistance changes, demonstrating that the PVA-APSi-EDTA_1_ hydrogel sensor can respond quickly and accurately to deformation. Figure 6g,h indicate that the sensor can sensitively detect subtle human activities like pulse beats and stably monitor flexing movements of the elbow and knee joints. Moreover, with consistent amplitude of human activity, the electrical signal remains on a stable plateau, proving its exceptional electrical signal stability. Additionally, the biocompatibility of the PVA-APSi-EDTA hydrogel was verified using the CCK-8 method to assess cytotoxicity to Huh-7 cells [41]. According to data from Figure S5, within 48 h, cell viability in both the dry and wet gel experimental groups remained above 90%, confirming the good biocompatibility of the PVA-APSi-EDTA hydrogel.

In summary, the PVA-APSi-EDTA_1_ hydrogel possesses outstanding mechanical properties, good biocompatibility, electrochemical stability, high sensitivity, and rapid response capabilities, making it suitable for real-time detection of human or mechanical movement and holding great potential in the field of flexible strain sensors.

### 2.6. Recycling of EOL Hydrogels

The recyclability of hydrogel materials is significant in reducing environmental pollution and resource waste. It effectively decreases energy consumption during the production process and enhances resource utilization efficiency [42]. The PVA-APSi-EDTA hydrogel exhibits excellent metal ion adsorption capacity in both single-metal and multi-element solutions. This can be primarily attributed to the hydrogel’s sponge-like porous structure, which facilitates the physical adsorption of metal ions. Additionally, the hydrogel contains functional groups such as –NH–, –NH_2_, and –COOH, which enable chemical adsorption of metal ions. In aqueous solutions, –COOH can dissociate into the negatively charged carboxylate ion (–COO^−^), causing the hydrogel surface to acquire a negative charge and thereby attract positively charged metal ions [43]. At the same time, the oxygen atoms of –COO^−^ and the nitrogen atoms in the amino groups possess lone pairs of electrons, which can form coordination bonds with metal ions, further enhancing the adsorption capacity. These two adsorption mechanisms work synergistically, significantly improving the hydrogel’s adsorption capacity and rate.

**Figure 7:**
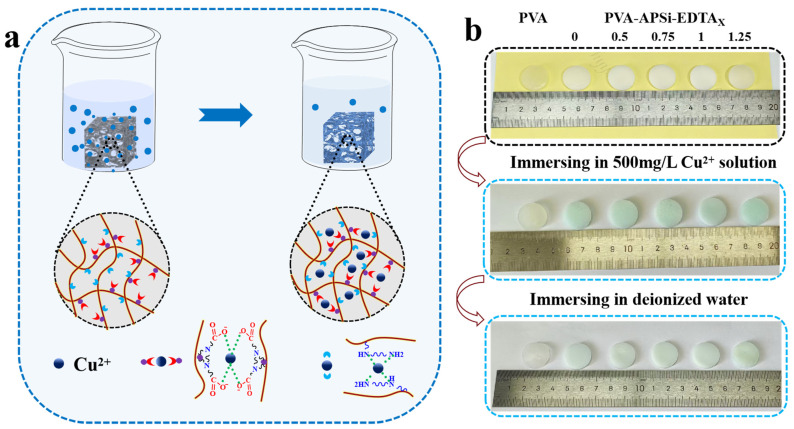
Adsorption behavior of hydrogels towards Cu^2+^. (**a**) Schematic diagram of the adsorption process. (**b**) Physical depiction of Cu^2+^ adsorption and post-soaking in water.

The PVA-APSi-EDTA hydrogel was cut into small pieces and placed in Cu^2+^ solutions of varying concentrations to simulate the Cu^2+^ adsorption performance of end-of-life (EOL) sensor waste (Figure 7a). For the hydrogel after Cu^2+^ adsorption, a deionized water soaking test showed that, apart from PVA hydrogel which could completely revert to its pre-adsorption state, the other five types of hydrogels still retained some of the adsorbed Cu^2+^, indicating that Cu^2+^ is firmly bound within the hydrogel matrix (Figure 7b). These results not only confirm the coexistence of physical and chemical adsorption within the hydrogel but also highlight the potential of PVA-APSi-EDTA hydrogel in environmental management applications.

**Figure 8:**
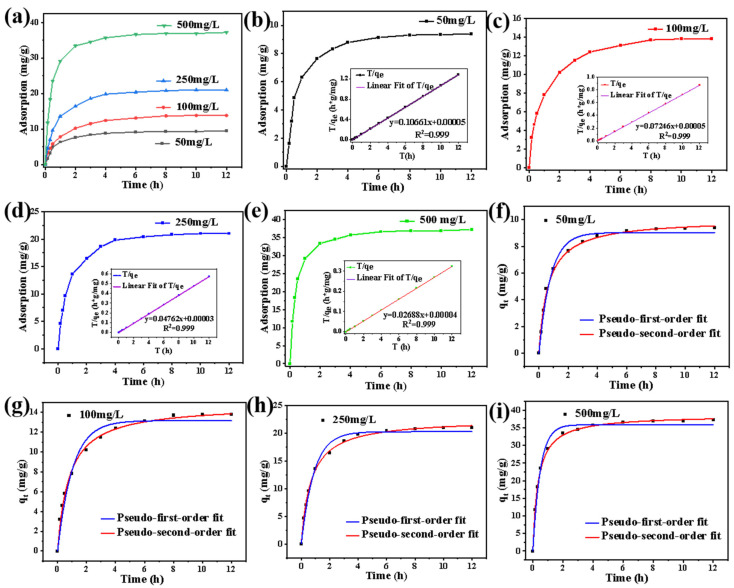
Adsorption kinetics of PVA-APSi-EDTA_1_ hydrogel. (**a**) Impact of initial Cu^2+^ concentration on adsorption. (**b**–**e**) Kinetic performance and pseudo-second-order linear fit model for adsorption of PVA-APSi-EDTA_1_ hydrogel at different initial Cu^2+^ concentrations: (**b**) 50 mg/L Cu^2+^, (**c**) 100 mg/L Cu^2+^, (**d**) 250 mg/L Cu^2+^, (**e**) 500 mg/L Cu^2+^. (**f**–**i**) Comparison of pseudo-first-order and pseudo-second-order kinetics for PVA-APSi-EDTA_1_ hydrogel at varying initial Cu^2+^ concentrations: (**f**) 50 mg/L, (**g**) 100 mg/L, (**h**) 250 mg/L, (**i**) 500 mg/L.

Adsorption rate and equilibrium adsorption capacity are critical indicators of adsorbent material performance. As shown in Figure 8a, the PVA-APSi-EDTA_1_ hydrogel rapidly reaches 80% of its equilibrium adsorption capacity within the first 2 h and fully achieves equilibrium after 8 h, demonstrating its efficient adsorption sites. Moreover, the equilibrium adsorption capacity is influenced by the initial concentration; as the initial Cu^2+^ concentration increases to 500 mg/L, the equilibrium adsorption capacity of the hydrogel reaches a maximum value of 37.27 mg/g. Figure 8b–e visually illustrate the impact of initial concentration on the adsorption performance of PVA-APSi-EDTA_1_ hydrogel, where the high fitting accuracy of the pseudo-second-order adsorption model with experimental data indicates that the adsorption behavior is primarily driven by chemical adsorption [44]. Comparisons between the pseudo-first-order and pseudo-second-order models for PVA-APSi-EDTA_1_ hydrogel at different initial concentrations (Figure 8f–i) reveal a higher R^2^ for the pseudo-second-order model (R^2^ > 0.995), further confirming that the adsorption behavior of PVA-APSi-EDTA_1_ hydrogel at any initial concentration is predominantly controlled by chemical adsorption [45,46]. Detailed data are available in Table S4.

The adsorption behavior for Cu^2+^ of the hydrogel is influenced by the presence of other metal ions and particles in the solution (Figure S6). For multi-metal ion adsorption, the adsorption capacity q_e_ is significantly lower than that for the single Cu^2+^ adsorption. This is due to the fact that Zn^2+^ and Fe^3+^ ions can interact with the negatively charged sites on the hydrogel surface, occupying the adsorption sites and reducing the adsorption of copper ions. Furthermore, Zn^2+^ and Fe^3+^ can form coordination complexes with the carboxyl and amino groups in the hydrogel, which further inhibits the adsorption of Cu^2+^. When Fe_2_O_3_ particles are present in the Cu^2+^ solution, the hydrogel’s ability to adsorb Cu^2+^ is suppressed. The surface of Fe_2_O_3_ particles has certain hydrophilic and charge properties, which allow them to compete with the hydrogel for binding sites, leading to a decrease in copper ion adsorption. Additionally, Fe_2_O_3_ particles may form coordination complexes with Cu^2+^ ions through surface reactions or complexation, thereby reducing the effective concentration of copper ions in the hydrogel and further lowering its adsorption capacity for Cu^2+^.

## 3. Conclusions

This study developed a hydrogel with outstanding comprehensive performance by constructing an APSi network within a PVA matrix. The hydrogel, through dense sacrificial bonds between networks, effectively dissipates energy, endowing it with high mechanical strength (1.46 MPa), high toughness (2.17 MJ/m^3^), and excellent elongation at break (398%). Additionally, the hydrogel also displays superior conductivity (10.97 S/cm) and high strain sensitivity (GF > 1), enabling precise detection of pulse beats, finger bending, and the flexing of elbow and knee joints. After 1000 cycle tests, the hydrogel also proved its remarkable stability. Moreover, at EOL, sensor waste can be utilized as a water treatment material for the adsorption of metal ions, achieving sustainable resource utilization.

This study presents a hydrogel with excellent mechanical properties, good biocompatibility, high strain sensitivity, and recyclability, demonstrating significant potential for applications in flexible strain sensors. Despite these advantages, certain limitations remain. The long-term stability of the hydrogel under high-strain conditions requires further optimization, especially when facing more complex application scenarios, where its durability and fatigue resistance may need to be enhanced. Moreover, the current fabrication methods still have room for improvement in terms of large-scale production and cost control. Future research could focus on optimizing the fabrication process of the hydrogel, improving its mechanical and electrical properties, while expanding its applications in smart wearable devices, health monitoring, and flexible electronics. Additionally, exploring the hydrogel’s biodegradability and biocompatibility would lay a more solid foundation for its widespread use in biomedical fields. Overall, this study demonstrates the enormous application potential of high-performance recyclable hydrogels in flexible strain sensors through a simple and cost-effective fabrication method, providing valuable theoretical support and experimental evidence for research and applications in related fields.

## 4. Materials and Methods 

### 4.1. Materials

All chemical reagents were used as received without further purification. Polyvinyl alcohol (PVA, type 1799, alcoholysis degree of 98–99%) was obtained from Shandong Ke yuan Biochemical Co., Ltd. (Shandong, China). N-(β-aminoethyl)-γ-aminopropyl methyldimethoxysilane (AEAPMDS) were purchased from Shanghai Aladdin Biochemical Technology Co., Ltd. (Shanghai, China). EDTA (AR, 99.5%) and FeCl_3_ (AR, 99%) were purchased from Shandong Keyuan Biochemical Co., Ltd. (Shandong, China); CuSO_4_·5H_2_O (conforming to standard SB 665–78) was purchased from Jinan Chemical Reagents Factory (Jinan, China); ZnCl_2_ (GR) was purchased from Shanghai Macklin Biochemical Co., Ltd. (Shanghai, China); and Fe_2_O_3_ (AR, 99.5%)was purchased from Shanghai Aladdin Biochemical Technology Co., Ltd. (Shanghai, China).

The hydrogel swelling solution, 5 g of PVA-APSi-EDTA_1_ hydrogel sample, was immersed in 10 mL of distilled water and soaked for 48 h. The hydrogel was then filtered out, and the remaining solution was evaporated and concentrated to 1 mL for future use.

### 4.2. Preparation of APSi

A total of 350 g of AEAPMDS and 70 g of deoxygenated distilled water (DI) were added to a 500 mL three-necked flask. The mixture was heated to 100 °C under a nitrogen atmosphere and maintained at this temperature for 2 h. Following this, the temperature was reduced to room temperature, and low-boiling-point impurities generated during the reaction were removed via vacuum suction. While maintaining the vacuum, the temperature was gradually increased to 170 °C and held for an additional 2 h to further eliminate impurities and facilitate the condensation of the reaction products. Throughout the process, the sample was continuously stirred mechanically at a speed of 180 revolutions per minute.

### 4.3. Preparation of PVA-APSi-EDTA Hydrogel

As depicted in Figure S1, the preparation method commenced by adding 6 g of PVA and x g of EDTA to a 100 mL three-necked flask containing (51-x) g of distilled water. The mixture was heated to 100 °C and mechanically stirred for 2 h. Subsequently, 3 g of APSi was added, and the temperature was maintained at 100 °C while stirring for an additional 2 h. Once the solution became homogeneous and transparent, it was allowed to cool, and the PVA-APSi-EDTAx precursor solution was extracted for centrifugation to remove air bubbles. Here, “x” represents the specific content of EDTA in the formulation. For example, PVA-APSi-EDTA_1_ indicates a sample with 1 g of EDTA in the formulation. The centrifuged precursor solution was then transferred into a container fitted with a PTFE membrane and subjected to three freeze–thaw cycles. Each cycle involved freezing at −20 °C for 21 h followed by thawing at room temperature for 3 h, to prepare the PVA-APSi-EDTAx hydrogel. Finally, the resulting hydrogel was soaked in distilled water for three days, with the water being changed every 12 h to remove any residual impurities.

### 4.4. Instruments and Methods

Fourier Transform Infrared Spectroscopy (FT-IR) (Bruker Corporation, Billerica, MA, USA): This was performed at room temperature on dried hydrogel samples using attenuated total reflection (ATR) mode to analyze changes in characteristic functional groups.

Nuclear Magnetic Resonance Spectroscopy (^1^H NMR) (Bruker Corporation, Billerica, MA, USA): APSi was dissolved in deuterated chloroform and analyzed at a frequency of 400 MHz to characterize its chemical structure and confirm its successful synthesis.

ACQUITY Advanced Polymer Chromatography (APC) (Agilent Technologies, Inc., Santa Clara, CA, USA): The molecular weight of APSi was determined using tetrahydrofuran as the mobile phase, at a flow rate of 4.0 mL/min, temperature of 40 °C, and a run time of 13 min.

Scanning Electron Microscopy (SEM) (JEOL Ltd., Tokyo, Japan) and Energy Dispersive Spectroscopy (EDS): Hydrogel samples were lyophilized and fractured in liquid nitrogen, with SEM used to characterize the microstructure of the hydrogel fractures, and EDS used to analyze the elemental composition content and distribution in the microregions of the hydrogel.

X-Ray Diffraction (XRD) (Bruker Corporation, Billerica, MA, USA): Characterization of lyophilized hydrogel samples was performed using a Bruker D8 Advance X-ray diffractometer.

Thermogravimetric Analysis (TGA) (Setaram Instrumentation, Lyon, France): This was performed under an air atmosphere, with a set heating rate of 10 °C/min and a test temperature range of 30 °C to 800 °C.

### 4.5. Water Content Tests

The degree of swelling (Sd) is calculated using the following formula:

(1) $$Sd=\frac{{W_{t}-W}_{d}}{W_{d}}$$

where W_t_ is the weight (g) of the hydrogel in a swollen state at time t, and W_d_ is the weight (g) of the freeze-dried hydrogel.

### 4.6. Mechanical Properties of the Hydrogel

The tensile and compression tests of the hydrogel samples were conducted using a universal testing machine (Tensile Testing Machine, Sansi Vertical Technology Co., Ltd., Shenzhen, China). In the tensile test, the hydrogel was shaped into a dumbbell form and subjected to a loading rate of 200 mm min^−1^ to measure the tensile strength and elongation at break, where strain (ε) is defined as the ratio of the extended length to the original length. For the loading–unloading test, the dumbbell-shaped samples were subjected to a constant loading rate of 50 mm min^−1^. The total area under the stress–strain curve is defined as toughness.

### 4.7. Electrical Properties of the Hydrogel

The conductivity (σ) of the hydrogel was measured using a handheld AC four-probe tester (Model ST2242, Suzhou Lattice Electronics Co., Ltd., Suzhou, China):

(2) $$\sigma \left(S/m\right)=\frac{1}{\rho }$$

(3) $$\rho \left(\Omega /m\right)=\rho_{0}*G\left(\frac{w}{s}\right)*D(\frac{d}{s})$$

where ρ is the resistivity, w is the sample thickness, s is the distance between the probes, d is the sample length, and G and D are the corresponding correction factors.

The electrical sensitivity (gauge factor, GF) of the hydrogel was measured using an electronic universal testing machine (Model ZQ-990LB, Zhi Qu Co., Ltd., Zhejiang, China),

(4) $$GF=\frac{R-R_{0}}{R*\varepsilon }$$

where R_0_ and R are the resistances without and with applied strain, respectively.

### 4.8. Adsorption Performance Testing

A stock solution of Cu^2+^ at a concentration of 1 g/L was prepared using CuSO_4_·5H_2_O and then diluted to the appropriate concentration prior to use. Fifty milliliters of the diluted Cu^2+^ solution was placed in a glass bottle, into which a water-swelled hydrogel, previously soaked in distilled water, was added. The mixture was stirred thoroughly at room temperature at a speed of 200 rpm. The remaining concentration of Cu^2+^ in the glass bottle was measured using a UV-Vis spectrophotometer (UV-4802) (Beijing Purkinje General Instrument Co., Ltd., Beijing, China). The adsorption kinetics of Cu^2+^ onto the hydrogel were studied using pseudo-first-order and pseudo-second-order kinetic models.

(5) $$q_{t}=q_{e}\left(1-e^{-k_{1}t}\right)$$

(6) $$\frac{1}{q_{t}}=\frac{1}{q_{e}}+\frac{1}{k_{2}\cdot {q_{e}}^{2}\cdot t}$$

Equations (5) and (6) represent the pseudo-first-order and pseudo-second-order adsorption kinetic models, respectively. In these equations, q_t_ denotes the amount of Cu^2+^ adsorbed by the hydrogel at time t (mg/g); q_e_ indicates the amount of Cu^2+^ adsorbed by the hydrogel when equilibrium is reached (mg/g); k_1_ is the rate constant for the pseudo-first-order adsorption kinetics (h^−1^); and k_2_ is the rate constant for the pseudo-second-order adsorption kinetics (h^−1^).

### 4.9. Adsorption Interference Test

The interference of mixed metal and particulate matter solutions containing Cu^2+^ on the adsorption of Cu^2+^ by the hydrogel was investigated. The initial concentration of all metal ions was 500 mg/L, and the concentration of particulates in the solution was also 500 mg/L. A 50 mL aliquot of each solution was placed into separate glass bottles, into which gel samples, pre-soaked in distilled water, were immersed. The bottles were then stirred at 200 rpm for 24 h at room temperature to ensure equilibrium adsorption was reached. The remaining concentration of Cu^2+^ in the glass bottles was measured using a UV-Vis spectrophotometer (UV-4802).

### 4.10. Biocompatibility of Hydrogel

Logarithmically growing Huh-7 cells were washed three times with PBS buffer, followed by digestion with 1 mL of trypsin for 5 min. The cells were resuspended in an appropriate amount of DMEM medium to obtain a cell suspension. This suspension was diluted to a concentration of 4 × 10^3^ cells/100 μL and added to two 96-well cell culture plates, with 100 μL of suspension per well, to form experimental and control groups. The plates were incubated in a cell culture incubator for 24 h.

The hydrogel was cut into small pieces, washed three times with PBS buffer, and irradiated with ultraviolet light for 5 h. It was then placed in incomplete DMEM medium at a ratio of 1 g/10 mL and incubated for 24 h. The supernatant was extracted and filtered for sterilization to obtain the hydrogel extract. The hydrogel extract was supplemented with 10% fetal bovine serum and 0.1% antibiotics and added to the experimental group cell culture plates, while an equal volume of complete DMEM medium was added to the control group cell culture plates.

A CCK-8 solution was prepared, and 10 μL of this solution was added to the 96-well plates. The plates were incubated in a 37 °C incubator for 0.5 to 4 h, and the absorbance at 450 nm and 630 nm was measured using a microplate reader (OD value = OD450 − OD630). The cell number was calculated based on the OD values, allowing for the assessment of hydrogel cytotoxicity.

### 4.11. Statistical Analysis

For strain sensor test data, regression analysis was applied to investigate the relationship between strain and sensor response. The resistance changes at different strain values were measured, and linear regression was used to calculate the sensor’s sensitivity. To further assess the accuracy and reliability of the sensor’s performance, the standard error and the coefficient of determination (R^2^) were also calculated to quantify the goodness of fit of the model. In the metal ion adsorption experiments, we applied adsorption kinetics models to fit the experimental data, analyzing the relationship between the adsorption capacity and the concentration of metal ions in order to evaluate the adsorption ability of the hydrogel material. All experiments were repeated more than three times to ensure the reliability and statistical significance of the results.

## Data Availability

The data supporting this article have been included as part of the Supplementary Materials.

## Supplementary Materials

The following supporting information can be downloaded at: https://www.mdpi.com/article/10.3390/gels10120788/s1, Figure S1: Synthesis process of APSi; Figure S2: (a) 1H NMR of APSi, (b) FT-IR of APSi, (c) GPC of APSi; Table S1: Composition and water content of the PVA-APSi-EDTA hydrogel; Figure S3: (a) 1H NMR of PVA-APSi-EDTA1 swelling solution, (b) FT-IR of PVA-APSi-EDTA1 swelling solution and distilled water; Figure S4: Total elemental composition of the sample. (a) PVA-APSi-EDTA1, (b) PVA-APSi-EDTA1.25; Table S2: Mechanical properties of the hydrogel; Table S3: Electrical properties of the hydrogel; Figure S5: Relationship between cell viability and culture time. (a,b) Cell viability after 24 h and 48 h of culture; Table S4: Adsorption pseudo-first-order and pseudo-second-order kinetic parameters of the hydrogel; Figure S6: Interference of Zn^2+^, Fe^3+^, and Fe_2_O_3_ particles with Cu^2+^ adsorption by PVA-APSi-EDTA_1_ hydrogel.
