# Supplementary material for: Silicon-Enhanced PVA Hydrogels in Flexible Sensors: Mechanism, Applications, and Recycling
Source: Gels. 2024 Dec 2;10(12):788. doi: 10.3390/gels10120788 (PMC11675336; doi:10.3390/gels10120788)
Supplement: Supplementary file 1 [file gels-10-00788-s001.zip › gels-3317060-supplementary.pdf]

Supporting Information

Silicon-Enhanced PVA Hydrogels, Applications in Flexible Sensors: Mechanism, Apply and Recycle

Xiaolei Guo[a], Hao Zhang[b], Manman Wu[a], Zhan Tian[a], Yanru Chen[a], Rui Bao[a], Jinghao Hao[a], Teng Long[a], Hua Wang[a], Xiao Cheng[a,c,*] and Chuanjian Zhou[a,c,*]

[a] Research Institute of Polymer Materials, School of Materials Science and Engineering, Shandong University, Jinan 250061, China;
[b] School of Materials Science and Engineering, University of Jinan, Jinan, Shandong, 250022 China;
[c]Weihai Research Institute of Industrial Technology, Shandong University, Weihai, 264209, China;
* Corresponding author: E-mail: chengxiao@sdu.edu.cn (X.C.); zhouchuanjian@sdu.edu.cn (C.Z.)

Content

Figure S1: Synthesis process of APSi

Figure S2: (a) ^{1}H NMR of APSi, (b) FT-IR of APSi, (c) GPC of APSi

Table S1: Composition and water content of the PVA-APSi-EDTA hydrogel

Figure S3: (a) ^{1}H NMR of PVA-APSi-EDTA$_1$ swelling solution, (b) FT-IR of PVA-APSi-EDTA1 swelling solution and distilled water.

Figure S4: Total elemental composition of the sample. (a)PVA-APSi-EDTA$_1$, (b)PVA-APSi-EDTA$_{1.25}$.

Table S2: Mechanical properties of the hydrogel

Table S3: Electrical properties of the hydrogel

Figure S5: Relationship between cell viability and culture time. (a, b) Cell viability after 24h and 48h of culture.

Table S4: Adsorption pseudo-first-order and pseudo-second-order kinetic parameters of the hydrogel

Figure S6: Interference of Zn^{2+}, Fe^{3+}, and Fe_2O_3particles with Cu^{2+} adsorption by PVA-APSi-EDTA$_1$ hydrogel.

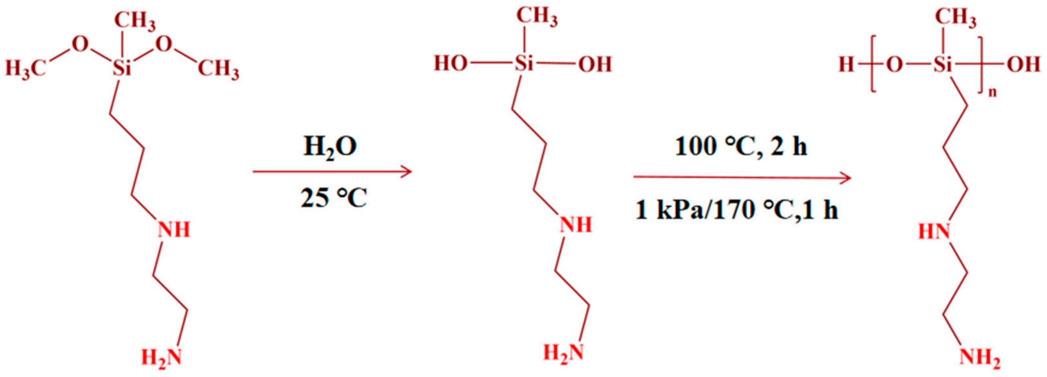

Figure S1 Synthesis process of APSi

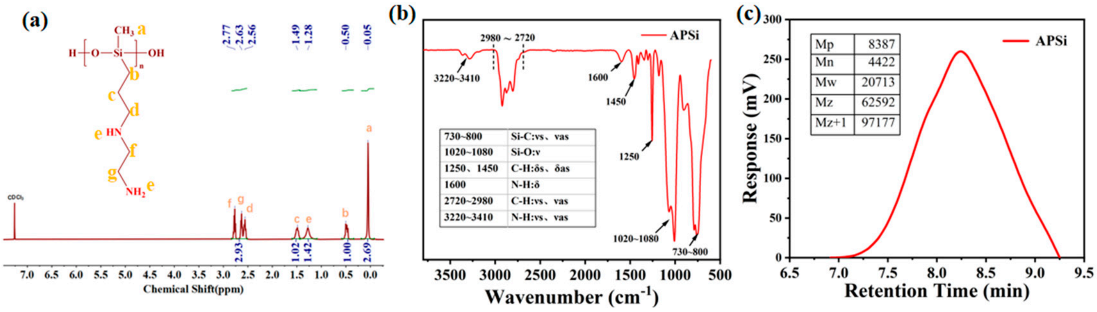

Figure S2 (a) ^{1}H NMR of APSi, (b) FT-IR of APSi, (c) GPC of APSi

Table S1 Composition and water content of the PVA-APSi-EDTA hydrogel

Hydrogels	PVA (g)	APSi (g)	EDTA (g)	DI (ml)	Water content (%)
**PVA**	**6**	**0**	**0**	**54**	**93.21**
**PVA-APSi-EDTA$_0$**	**6**	**3**	**0**	**51**	**88.53**
**PVA-APSi-EDTA$_{0.5}$**	**6**	**3**	**0.5**	**50.5**	**86.89**
**PVA-APSi-EDTA$_{0.75}$**	**6**	**3**	**0.75**	**50.25**	**87.64**
**PVA-APSi-EDTA$_1$**	**6**	**3**	**1**	**50**	**86.72**
**PVA-APSi-EDTA$_{1.25}$**	**6**	**3**	**1.25**	**49.75**	**87.44**

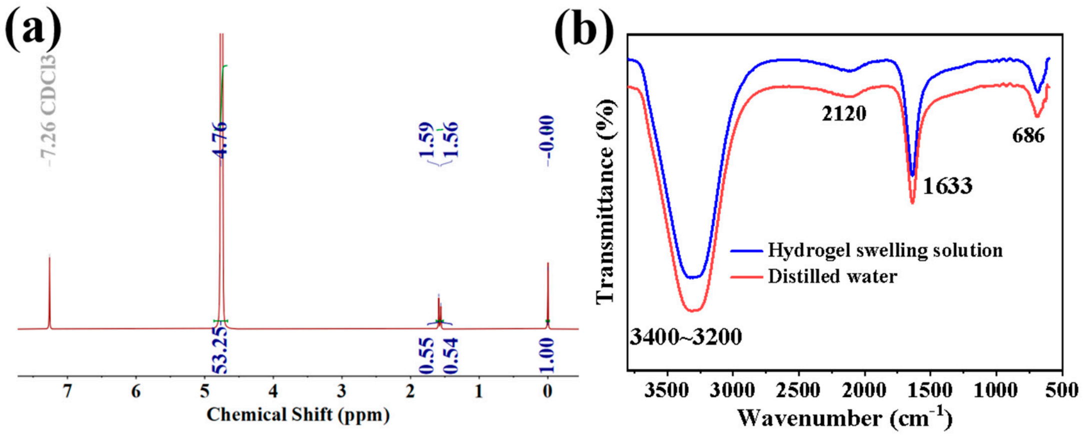

Figure S3 (a) ^{1}H NMR of PVA-APSi-EDTA$_1$ swelling solution, (b) FT-IR of PVA-APSi-EDTA$_1$ swelling solution and distilled water.

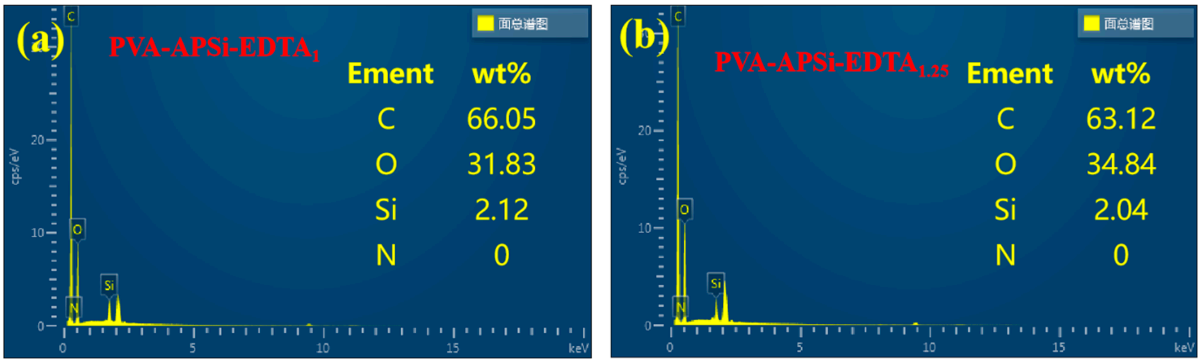

Figure S4 Total elemental composition of the sample. (a)PVA-APSi-EDTA$_1$, (b)PVA-APSi-EDTA$_{1.25}$.

Table S2 Mechanical properties of the hydrogel

Hydrogels	Tensile strength (MPa)	Elongation (%)	Toughness (MJ/m^3)
PVA	0.071	216	0.058
PVA-APSi-EDTA$_0$	0.461	243	0.436
PVA-APSi-EDTA$_{0.5}$	0.659	331	0.803
PVA-APSi-EDTA$_{0.75}$	1.054	353	1.423
PVA-APSi-EDTA$_1$	1.461	357	2.170
PVA-APSi-EDTA$_{1.25}$	0.953	397	1.361

Table S3 Electrical properties of the hydrogel

Hydrogels	Conductivity (S/cm)	Gauge factor (GF)	Correlation coefficient (r)	Linear coefficient (R^2)	Linear fitting curve
PVA	0.110	GF=2.093	r=0.997	R^2=0.995	y=2.093x-8.042
PVA-APSi-$EDTA_0$	0.820	GF=0.922	r=0.997	R^2=0.994	y=0.922x-4.213
PVA-APSi-$EDTA_{0.5}$	3.133	GF=1.436	r=0.998	R^2=0.996	y=1.436x-4.247
PVA-APSi-$EDTA_{0.75}$	7.033	GF=1.263	r=0.999	R^2=0.998	y=1.263x-3.049
PVA-APSi-$EDTA_1$	10.967	GF=1.425	r=0.999	R^2=0.998	y=1.425x-4.224
PVA-APSi-$EDTA_{1.25}$	8.067	GF=1.083	r=0.998	R^2=0.997	y=1.083x-2.909

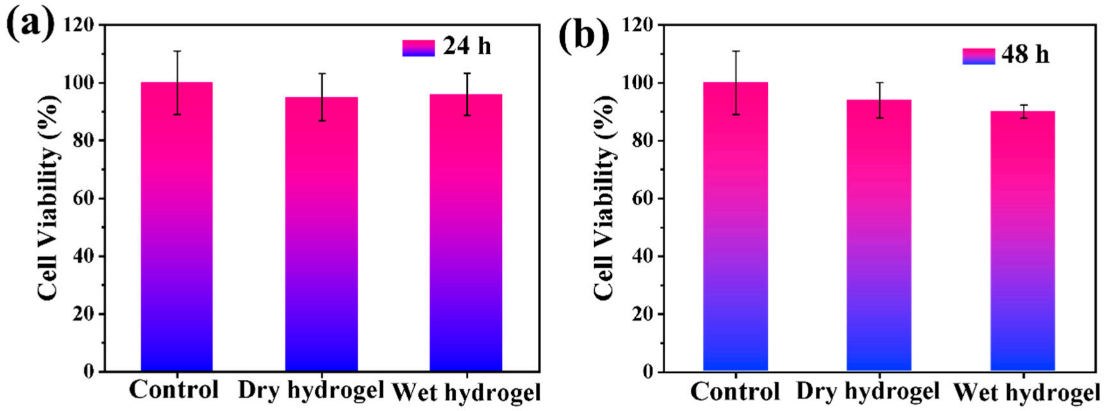

Figure S5 Relationship between cell viability and culture time. (a, b) Cell viability after 24h and 48h of culture.

Table S4 Adsorption pseudo-first-order and pseudo-second-order kinetic parameters of the hydrogel

C_0 (mg/L)	q_{e1} (mg/g)	k_1 (h^{-1})	R_1^2	q_{e2} (mg/g)	k_2 (h^{-1})	R_2^2
50	9.039	1.250	0.988	10.046	0.158	0.995
100	13.168	0.968	0.974	14.771	0.085	0.996
250	20.264	1.136	0.987	22.573	0.065	0.998
500	35.865	2.047	0.990	38.627	0.074	0.998

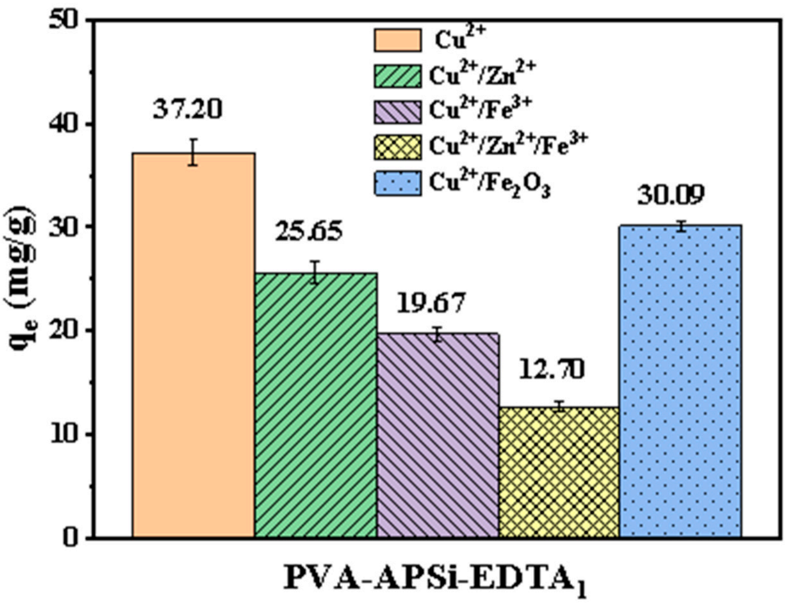

Figure S6 Interference of Zn^{2+}, Fe^{3+}, and Fe_2O_3 particles with Cu^{2+} adsorption by PVA-APSi-EDTA$_1$ hydrogel.
